# Unlocking the Microbial World: A Global Initiative for Education and Sustainability

**DOI:** 10.1111/1751-7915.70124

**Published:** 2025-04-07

**Authors:** Juan L. Ramos, Rup Lal, Francisca Colom, Max Chavarria, Wei Huang, Yun Wang, Zulema Udaondo, Kenneth N. Timmis

**Affiliations:** ^1^ Estación Experimental del Zaidín (EEZ) CSIC Granada Spain; ^2^ Acharya Narendra Dev College University of Dehli New Dehli India; ^3^ Laboratory of Medical Mycology Universidad Miguel Hernández Alicante Spain; ^4^ Escuela de Química y Centro de Investigación en Productos Naturales (CIPRONA) Universidad de Costa Rica San José Costa Rica; ^5^ Centro Nacional de Innovaciones Biotecnológicas (CENIBiot), CeNAT‐CONARE San José Costa Rica; ^6^ Department of Engineering Science University of Oxford Oxford UK; ^7^ Oxford Suzhou Center for Advanced Research Suzhou Jiangsu China; ^8^ Centro Nacional de Biotecnología (CNB), CSIC Madrid Spain; ^9^ Division of Microbiology Technical University of Braunschweig Braunschweig Germany

**Keywords:** education, microbial biotechnology, topic framework

## Abstract

Microbes govern our planet! The International Microbial Literacy Initiative (IMiLI) promotes global microbial literacy with free, open‐access resources in multiple languages. Understanding microbes is key to sustainability and informed decision‐making.

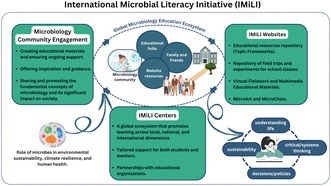

The Earth's biosphere is a microbial world driving essential geochemical processes and influencing all life, including humans. Microbes have shaped planetary chemistry, enabling evolution from prokaryotes to eukaryotes. In essence, microbes govern the planet. Therefore, understanding microbes is vital for understanding our world, our role in the biosphere and how to live sustainably with other organisms. Achieving this requires a sustained global effort by dedicated institutions.

The IMiLI (International Microbial Literacy Initiative) Project that was launched in 2019 (Timmis et al. 2019) primarily aims at promoting microbial literacy, emphasising practical knowledge of microbial activities and their impact on the biosphere, society and individuals. This understanding supports informed decision‐making on personal and global issues and is considered essential education for all (Timmis et al. 2024; Timmis et al. 2019; Anand et al. 2023).

IMiLI's primary goal is to create free, open‐access microbiology resources (CC BY‐NC 4.0) for learners worldwide. These resources highlight microbial concepts in the context of sustainability, social responsibility and decision‐making, developed collaboratively by microbiologists and experts from various fields. Originally created in English, these resources are being translated and adapted to cultural contexts by regional centres, including IMiLI‐SAC (South Asia Centre‐https://imili.org/); IMiLI‐CAC (Central America and the Caribbean, https://www.chavarrialab.com/imilicac) IMiLI‐EAH (East‐Asia Headquarters, http://www.imili‐eah.com) and Spain (https//grupos.eez.csic.es/imili/). These centres also collaborate with educators, serving as key educational hubs. Building on the development of hundreds of such materials, IMiLI has now launched an initiative to translate them into multiple languages, including Hindi, Spanish, French and Chinese. This endeavour aims to enhance microbial literacy by making scientific knowledge more accessible to diverse linguistic communities, thus promoting a deeper understanding of microbial sciences across different cultures and regions. To achieve this, regional centres are actively organising teacher workshops, school talks and experimental activities using available resources (e.g., https://imili.org/outreachprogram.php, https://imili.org/Symposiums.php, https://www.chavarrialab.com/imiliactivities).

IMiLI educational materials focus on engaging learners through relevant and inspiring topics, emphasising applications of microbial activities in promoting health and environmental sustainability (Van Beek et al. 2024). While schoolchildren and educators are the primary audience, IMiLI resources are designed to be accessible to people of all ages and societal backgrounds, fostering a global microbiology lifelong education ecosystem. These materials are written in a simple and engaging style, ensuring that even those with no prior knowledge of microbiology can easily grasp the significance of microbes in environmental protection, climate change and human health. By making complex scientific concepts understandable to a wider audience. IMiLI aims to cultivate microbial literacy and inspire a deeper appreciation for the role of microorganisms in sustaining life on Earth.

IMiLI's educational resources include:

1. Topic frameworks (TFs): A curriculum of over 200 knowledge frameworks grouped into 20 categories, adaptable for various teaching objectives and age groups. Another 200 are in the pipeline.
2. MicroStars portrait gallery: Illustrated ‘halls of fame’ for microbes driving key processes, helping students form memorable associations.
3. Class excursions: Practical, local experiences connecting everyday observations to microbial activities (McGenity et al. 2020).

Further resources, such as the following, are in the pipeline or at the planning stage:

4 Multi‐media educational materials (MATs): Interactive resources such as videos, animations, comics and games tailored to enhance engagement and clarity (Van Beek et al. 2024).
5 Classroom experiments: Activities immersing students in microbial processes.
6 Virtual fieldwork: Exploration of exotic microbiological research sites, inspiring a sense of adventure and discovery.
7 Homework and projects: Independent exploration and dissemination of microbial topics through creative and relatable assignments.

IMiLI envisions a global education ecosystem in microbiology supported by an international curriculum, a community of microbiologists and interconnected regional centres. Together, they aim to equip people worldwide with critical knowledge for societal and biosphere well‐being. By making microbiology resources readily available online, IMiLI seeks to influence educators, administrators and policymakers to recognise the critical role of microbial literacy. This initiative could eventually lead to microbiology being established as a dedicated subject in schools, the allocation of specific funding for its promotion, and its inclusion as a compulsory subject. By integrating microbial education into mainstream curricula, IMiLI aspires to foster a deeper public understanding of microbes and their fundamental role in environmental sustainability, climate resilience and human health.

IMiLI's current position results from a collective effort involving more than 700 scientists worldwide and the establishment of three regional centres, with a fourth in development. Others are in discussion. Each centre plays a crucial role in advancing microbial literacy. While this marks a significant milestone, we acknowledge that it is only the beginning of a much longer journey. The success and expansion of this initiative require sustained commitment and collaboration from the broader scientific community—not only microbiologists, biochemists and geneticists but also educators, policymakers, administrators and global leaders.

Achieving IMiLI's objectives calls for a multidisciplinary approach, where scientific expertise converges with societal engagement and policy‐driven action. By fostering international partnerships and institutional support, we can ensure that microbial education can reach every corner of the world, empowering individuals with the knowledge necessary to tackle global challenges in health, the environment and climate change.

This editorial and the special issue serve as an important reminder that while progress has been made, much work remains. The message is clear: every contribution, no matter how big or small, is valued and essential in advancing microbial literacy worldwide.

We strongly encourage researchers, educators, policymakers and all interested individuals to share new insights, propose innovative ideas and actively contribute to ongoing IMiLI initiatives. Whether through research, education, advocacy or policy development, every effort plays a role in achieving IMiLI's mission.

Furthermore, we invite you to engage directly with the authors of this editorial, who represent the IMiLI community. Your suggestions, feedback and collaborative efforts are invaluable in shaping the future of microbial education and ensuring that this initiative continues to expand and evolve to meet global challenges.

## Author Contributions

**Francisca Colom:** conceptualization, writing – original draft, writing – review and editing. **Juan L. Ramos:** writing – original draft, conceptualization, writing – review and editing. **Kenneth N. Timmis:** conceptualization, writing – original draft, writing – review and editing. **Max Chavarria:** writing – original draft, writing – review and editing, conceptualization. **Rup Lal:** writing – original draft, writing – review and editing, conceptualization. **Wei Huang:** conceptualization, writing – original draft, writing – review and editing. **Yun Wang:** conceptualization, writing – original draft, writing – review and editing. **Zulema Udaondo:** conceptualization, writing – original draft, writing – review and editing.

## Conflicts of Interest

The authors declare no conflicts of interest.

## Data Availability

Data sharing not applicable to this article as no datasets were generated or analysed during the current study.
